# Supplementary material for: Higher CCL22+ Cell Infiltration is Associated with Poor Prognosis in Cervical Cancer Patients
Source: Cancers (Basel). 2019 Dec 12;11(12):2004. doi: 10.3390/cancers11122004 (PMC6966573; doi:10.3390/cancers11122004)
Supplement: Supplementary file 1 [file cancers-11-02004-s001.pdf]

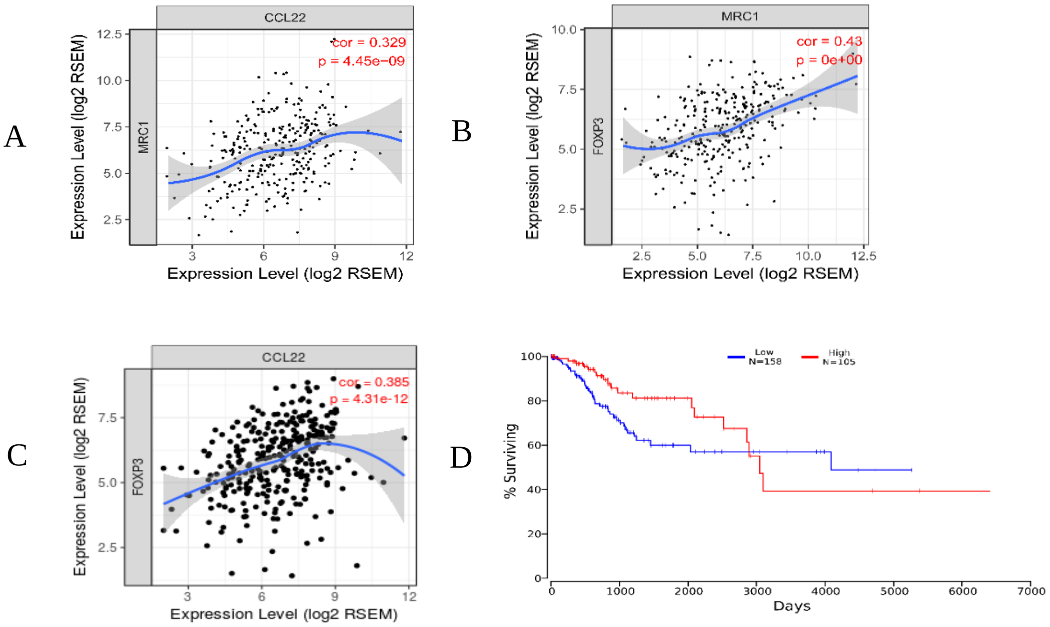

S1: Correlation analysis of CCL22, MRC1 and FOXP3 in CESC tissue as explored by the TIMER database. Cor, correlation coefficient. B. Survival analysis for high and low expression groups of CCL22. Red and blue colors denote the high and low expression of CCL22, respectively. CESC, cervical squamous cell carcinoma and endocervical adenocarcinoma
